# High-Entropy Perovskite Thin Film in the Gd-Nd-Sm-La-Y-Co System: Deposition, Structure and Optoelectronic Properties

**DOI:** 10.3390/ma16124210

**Published:** 2023-06-06

**Authors:** Pawel A. Krawczyk, Wojciech Salamon, Mateusz Marzec, Michał Szuwarzyński, Jakub Pawlak, Jarosław Kanak, Małgorzata Dziubaniuk, Władyslaw W. Kubiak, Antoni Żywczak

**Affiliations:** 1 Faculty of Materials Science and Ceramics, AGH University of Science and Technology, Al. Mickiewicza 30, 30-059 Kraków, Poland; dziubani@agh.edu.pl (M.D.); kubiak@agh.edu.pl (W.W.K.); 2 Academic Centre for Materials and Nanotechnology, AGH University of Science and Technology, Al. Mickiewicza 30, 30-059 Kraków, Poland; salamon@agh.edu.pl (W.S.); marzecm@agh.edu.pl (M.M.); szuwarzy@agh.edu.pl (M.S.); jakub.pawlak@agh.edu.pl (J.P.); 3 Institute of Electronics, AGH University of Science and Technology, Al. Mickiewicza 30, 30-059 Kraków, Poland; kanak@agh.edu.pl

**Keywords:** thin films, entropy-stabilized perovskites, pulsed laser deposition, fused quartz substrate

## Abstract

Multicomponent equimolar perovskite oxides (ME-POs) have recently emerged as a highly promising class of materials with unique synergistic effects, making them well-suited for applications in such areas as photovoltaics and micro- and nanoelectronics. High-entropy perovskite oxide thin film in the (Gd_0.2_Nd_0.2_La_0.2_Sm_0.2_Y_0.2_)CoO_3_ (RECO, where RE = Gd_0.2_Nd_0.2_La_0.2_Sm_0.2_Y_0.2_, C = Co, and O = O_3_) system was synthesized via pulsed laser deposition. The crystalline growth in an amorphous fused quartz substrate and single-phase composition of the synthesized film was confirmed by X-ray diffraction (XRD) and X-ray photoelectron spectroscopy (XPS). Surface conductivity and activation energy were determined using a novel technique implementing atomic force microscopy (AFM) in combination with current mapping. The optoelectronic properties of the deposited RECO thin film were characterized using UV/VIS spectroscopy. The energy gap and nature of optical transitions were calculated using the Inverse Logarithmic Derivative (ILD) and four-point resistance method, suggesting direct allowed transitions with altered dispersions. The narrow energy gap of RECO, along with its relatively high absorption properties in the visible spectrum, positions it as a promising candidate for further exploration in the domains of low-energy infrared optics and electrocatalysis.

## 1. Introduction

The demand for cutting-edge technology requires the development of new materials, which meet the ever-increasing requirements for conductivity, thermal stability, durability, and environmental resilience. In response to this need, high-entropy materials (HEMs) have garnered widespread attention, particularly the pioneering class of high-entropy alloys (HEAs) known for their remarkable versatility [1]. In 2015, expanding upon the concept of HEAs, Rost et al. [2] introduced a novel approach to ionic compounds by populating cation positions with several elements in equiatomic amounts. This breakthrough led to the emergence of a new class of materials called high-entropy oxides (HEOs), which encompass later, more complex structures, including spinel- [3] and perovskite-type oxides [4]. HEOs have gained significant attention from the research community due to their exceptional properties, attributed in part to the cocktail effect [5,6]. The thermodynamic stability of these materials is achieved by minimizing the Gibbs free energy. In the context of HEOs, phase stabilization relates to the Gibbs free energy Equation (1),

(1) ΔG=ΔH − TΔS 

where ∆G represents the change in Gibbs free energy, ∆H is the change of enthalpy, T is the absolute temperature, and ∆S is the change of entropy of the system [7]. The calculation of ∆S, which plays a crucial role in stabilizing the single-phase structure in high-entropy materials, is based on Equation (2),

(2) $$\Delta S=-R\;\sum_{i=1}^{\;N}x_{i}{\mathrm{lnx}}_{i}$$

where R is the universal gas constant and x_i_ is the molar concentration of each element in the mixture [7,8]. For instance, the entropy of HEOs consisting of five metal ions in the cationic sublattice is equal to 1.61 R [9].

Since the discovery of HEOs, the majority of research efforts have been directed toward bulk HEO systems, with relatively less attention given to thin film developments. The first reports on high-entropy oxide films (HEOFs) appeared in 2016, when Rost et al., [10] successfully prepared the entropy-stabilized oxide film using pulsed laser deposition (PLD), thus paving the way for further exploration [11]. Notably, perovskite thin films have garnered special attention due to their tunable physical and chemical properties, as evidenced by the recent studies on thermoelectric behavior, quantum effects, and structural and optical controlling [12,13,14,15]. The combination of various effects in a single material through 2D nanostructuring, exploiting the properties of the ABO_3_ family of perovskites and harnessing the synergistic effects of high configuration entropy has attracted significant interest. This approach promises the occurrence of pioneering physical phenomena, such as colossal magnetoresistance and photo- and electrocatalytic properties, alongside the simultaneous presence of emerging phases [16,17,18,19,20]. Consequently, these led to a growing focus on the fabrication of the high-entropy perovskite thin films used in a wide spectrum of fields, including nanoelectronics [21,22], electrocatalysis [23,24], and photovoltaics [25,26]. Moreover, the high-entropy effect resulting from the general configurational disorder of HEMs can effectively modulate the structural disorder, enabling access to unexplored quantum phase spaces and facilitating fundamental studies on the electronic band structure of those multicomponent materials [27,28,29].

The reliable production of uniform thin films on the planar crystalline and amorphous substrates via pulsed laser deposition is crucial for the development of cutting-edge applications, including artificial intelligence, memory, and logic devices [30,31,32,33,34,35,36,37]. One of the principal challenges encountered in thin film deposition processes for complex multicomponent oxide systems is the inherent risk of losing precise control over the stoichiometry of the film. Deviations from the desired composition can lead to the formation of secondary phases and hinder the attainment of a single-phase structure, which is particularly critical in multicomponent perovskite-type oxide systems [38]. Furthermore, the formation of defects, such as oxygen vacancies, during the film growth process can negatively impact film properties and stability [39]. To address these issues, the optimization of the deposition conditions, such as substrate temperature and oxygen pressure, becomes crucial in minimizing defect formation and enhancing film quality [40]. Motivated by the aforementioned potential, we have undertaken the challenge of nanostructuring the high-entropy perovskite-type oxide system that was the subject of our previous work [41].

This study aimed to deposit a thin film from the RECO system (Gd_0.2_Nd_0.2_La_0.2_Sm_0.2_Y_0.2_; C = Co; O = O_3_) onto a SiO_2_ substrate using pulsed laser deposition in order to investigate the feasibility of producing uniform films of a high-entropy multicomponent single-phase material. Our focus was primarily on the comprehensive characterization of the structural, chemical, electrical, and optical properties of the RECO thin film, intending to identify potential applications for this material.

## 2. Materials and Methods

*Target and thin film deposition:* The selection of the RECO (RE = Gd_0.2_Nd_0.2_La_0.2_Sm_0.2_Y_0.2_; C = Co; O = O_3_) for this study was based on the successful synthesis of this system, as detailed in our previous work [41]. The selection of this specific configuration was primarily driven by the requirement of matching the oxidation state and coordination number with the prototype structure of LaCoO_3_ in the *Pnma* space group. Additionally, the similarity in ionic radii of cations on a specific site (A or B) was quantitatively examined using the tolerance factor introduced by Goldschmidt for perovskite-type oxides [42] and the parameter δ proposed by Zheng for high-entropy materials [43], yielding values of 0.932 [−] and 0.344 %, respectively. The thin film has been deposited on a fused quartz (SiO_2_) substrate employing the pulsed laser deposition (PLD) technique. The KrF 248 nm excimer laser (Compex Pro 110) with a pulse of 10 ns and energy density of 1.5 J cm^−2^ has been used for the ablation-deposition processes. The distance between the rotating substrate holder and the RECO target was set to 5 cm. All other parameters, i.e., substrate temperature of 750 °C, deposition rate of 10 Hz and 5000 pulses, and oxygen background pressure of 40 mTorr, have resulted in a thin film with a thickness of approximately 100 nm. The parameters were optimized based on work on the deposition of a thin film of perovskite with a similar structure, namely LaCoO_3_, as described in reference [44], and tuned by further testing of the RECO target material in the PLD process.

*AFM:* Topography and conductive parameters of the obtained thin film were studied with atomic force microscopy (AFM). Images were obtained with a Dimension Icon XR (Bruker, Santa Barbara, CA, USA) working in the PeakForce Tapping (PFT) mode in the air using Platinum-Iridium (Pt/Ir) front-side-coated, electrically conductive tips with a nominal spring constant of 3 N·m^−1^ and nominal radius of curvature of 25 nm. PeakForce TUNA mode was used for conductivity imaging with a bias voltage equal to 10 V and for collecting I–U curves from the random spots on (Gd_0.2_Nd_0.2_La_0.2_Sm_0.2_Y_0.2_)CoO_3_ surfaces. I–U plots for all samples were collected in the same conditions using ramp mode with a setpoint value corresponding to the maximum force of 1 nN and the voltage applied between the tip and the sample surface in the range −5 V to 5 V. The plots were recorded in at least 25 random points for each sample. Topography and conductive images with the corresponding I−U plots were obtained at room temperature (298 K) and elevated temperatures (323 K, 348 K, and 373 K). All elevated temperatures measurements were done on the High Temperature Heater element from Bruker Co. (Santa Barbara, CA, USA) added to the AFM stage and cooled by the flow of chilled water driven by the Masterflex L/S peristaltic pump (Cole Parmer, Vernon Hills, IL, USA). The temperature values were set by the Thermal Application Controller (Bruker) driven by the software (Nanoscope 9.7) and controlled by an external thermometer connected directly to the sample surface. All measurements were done in the environmental chamber providing thermal and acoustic insulation.

*XRD:* X-ray diffraction studies were made through an X’Pert MPD diffractometer equipped with CuKα radiation. The substrate and target prepared according to the procedure described in our previous study [41] were measured in Bragg-Brentano and grazing incident X-ray diffraction (GIXD) geometry at an incidence angle of 1°. Thin film thickness and interface roughness were obtained via X-ray reflectivity (XRR) measurements.

*XPS:* X-ray photoelectron spectroscopy analyses were carried out in a PHI Versa ProbeII Scanning XPS system using monochromatic Al Kα (1486.6 eV) X-rays focused to a 100 µm spot and scanned over the area of 400 µm × 400 µm. The photoelectron take-off angle was 45° and the pass energy in the analyzer was set to 46.95 eV to obtain high energy resolution spectra for the Co 2p, O 1s, Sm 3d, Nd 3d, Gd 4d, La 3d, Y 3d, and Bi 4f regions. A dual beam charge compensation with 7 eV Ar^+^ ions and 1 eV electrons was used to maintain a constant sample surface potential regardless of the sample conductivity. All XPS spectra were charge referenced to the unfunctionalized, saturated carbon (C−C) C1s peak at 285.0 eV. The operating pressure in the analytical chamber was less than 3·10^−9^ mbar. Deconvolution of spectra was carried out using PHI MultiPak software (v.9.9.0.8). Spectrum background was subtracted using the Shirley method. The Lorentzian-Gaussian function was employed for fitting, with the Gaussian part accounting for at least 80% of the curve shape.

*Conductivity measurements*: Resistivity of RECO on SiO_2_ was measured using the standard four-point resistance method (FPRM) with a Keithley model 2400 source meter unit. To determine the activation energy of the obtained material, the temperature range of 340–450 K at intervals of 10 K was applied. Measurements were made in an argon atmosphere to ensure high stability and to prevent external contamination of the sample.

*UV/VIS measurements*: Optical measurements of RECO/quartz film were made with a 1 nm step using the PerkinElmer UV–Vis Lambda 750 spectrophotometer.

## 3. Results and Discussion

**Figure 1:**
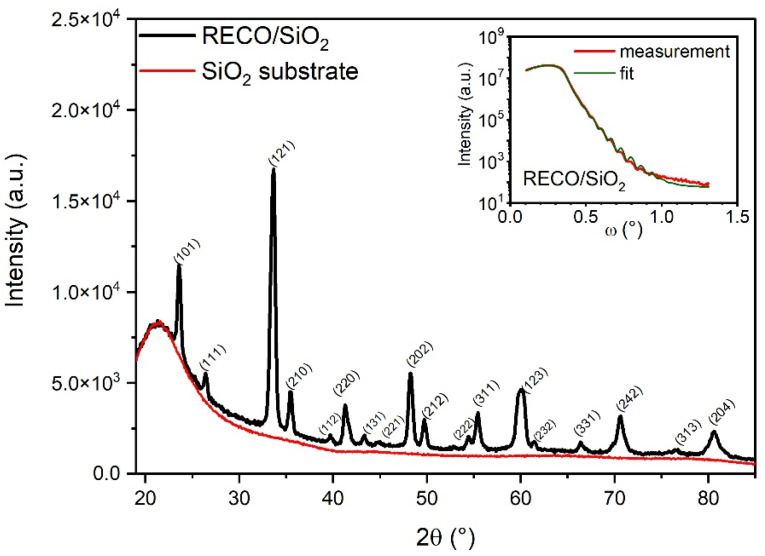
X-ray diffraction pattern of RECO thin film deposited onto a fused quartz (SiO_2_) substrate. Inset shows a plot with fitting and experimental X-ray reflectivity profile for RECO thin film layer on a quartz substrate.

Figure 1 shows the grazing incident X-ray diffraction (GIXD) profile for a thin RECO film (black line) that was deposited onto a SiO_2_ (fused quartz) substrate surface. The red profile corresponds to a measurement taken from an uncovered substrate, revealing its amorphous structure. The relatively high Full Width at Half Maximum (FWHM) of the RECO reflections is attributed to the limited thickness of the film. Additionally, the X-ray reflectivity profile of the RECO layer on quartz is presented in the inset of Figure 1. The density, layer thickness, and RMS roughness were determined through an optimal match between the experimental curve (Figure 1: red, measurement) and the calculated XRR profile (Figure 1: green, fit). The values for density, layer thickness, and roughness are presented in Table 1.

The XRD pattern obtained for the RECO thin film presents a good fit with the orthorhombic group of the *Pnma* symmetry (as indexed in ICSD 01-086-1208), which is consistent with our previous work on bulk RECO material [41]. The presence of distinct peaks in the XRD profile coming from different crystallographic planes with a pattern with the space group *Pnma* supports the conclusion of disoriented crystallite growth on the amorphous SiO_2_ substrate. This observation equivalently indicates the absence of epitaxial growth, which can be attributed to the lack of a regular lattice structure, and the inability to induce a preferred crystallographic orientation on the RECO thin film [45]. The interplanar spacing calculated from the position of the peaks is smaller than 0.2% compared to the RECO bulk powder sample. Grain sizes were determined by applying the Debye Scherrer formula to the Full Width at Half Maximum (FWHM) values of the peaks [46]. The calculated grain sizes for these peaks range from 13 to 20 nm. These results suggest that the structure and properties of the RECO thin film may differ from those of the bulk material.

**Figure 2:**
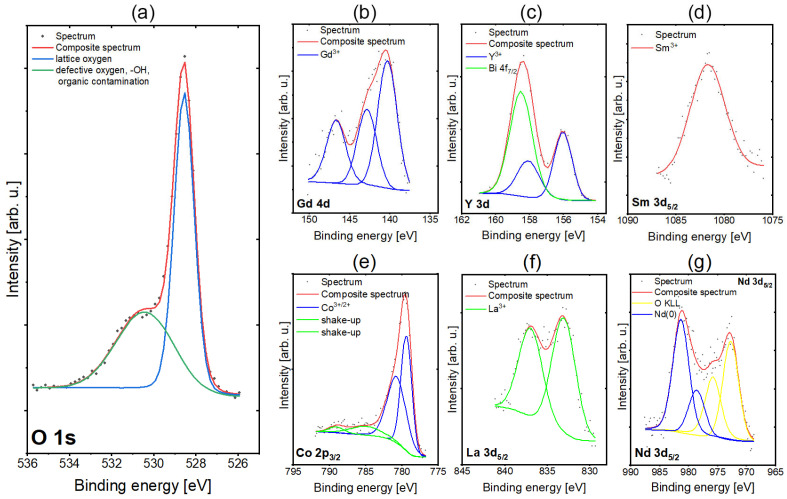
High-resolution individual XPS spectra of RECO thin films for binding energies and chemical states and peak fitting for (**a**) oxygen, (**b**) cobalt, (**c**) samarium, (**d**) gadolinium, (**e**) neodymium, (**f**) lanthanum and (**g**) yttrium.

X-ray photoelectron spectroscopy (XPS) has been used to investigate the surface chemistry of the deposited RECO thin film. The O 1s spectrum depicted in Figure 2a shows two peaks—the first one at 528.5 eV originating from lattice oxygen in metal oxides and the second one at 531.1 eV, which comes from either defective oxygen or organic contamination [47]. The XPS Co 2p_3/2_ region spectra (Figure 2b) are similar and show multiplet splitting structure characteristics for first-row transition metal species containing unpaired electrons. From the inspection of line positions (first peak centered at 779.5 eV) and the very low intensity of shake-up satellites, it can be stated that cobalt is in Co^3+^ and Co^2+^ oxidation states such as in Co_3_O_4_ [47,48,49]. The XPS Sm 3d_5/2_ spectrum is shown in Figure 2c. The Sm 3d_5/2_ core level peak maximum was found at 1081.5 eV, indicating the literature value characteristic for samarium oxidation state Sm^3+^ [50]. The XPS Gd 4d spectrum is depicted in Figure 2d. From inspection of the fitted line positions (main peak at 140.5 eV) and the spectrum shape, it can be stated that gadolinium exists predominantly in the Gd^3+^ oxidation state, as observed in gadolinium oxide (Gd_2_O_3_) [51]. The Nd 3d_5/2_ spectra are shown in Figure 2e. The observed overlap between the peaks of neodymium (Nd) and the Auger oxygen (O) KLL lines was taken into consideration during the fitting process. Despite this overlap, the Auger lines were successfully extracted from the data. Nd 3d_5/2_ lines were found at about 980 eV, indicating the metallic state of the neodymium [52]. The La 3d_5/2_ spectra presented in Figure 2f were fitted with two components. In the case of lanthanum (La), only one chemical state is observed. However, due to the phenomenon of multiplet splitting, each state manifests as two distinct lines. The assigned chemical state for lanthanum (La) was determined to be La2O3 based on the observed lines at the binding energies of 832 and 837 eV [50,51]. Deconvolution of spectra collected for Y 3d regions results in lines centered at positions typical for Yttrium in the Y^3+^ oxidation state, similar to that found in YSZ (Figure 2g) [50]. The presence of bismuth has been detected in the spectrum of yttrium, and from the position of the line of Bi 4f_7/2_, it can be determined that bismuth is in the oxidation state of Bi^3+^ [50,51]. The bismuth traces identified on the surface can be attributed to the contamination from the pulsed laser deposition chamber, which was instinctively used to prepare BiFeO_3_ layers before RECO/SiO_2_ film deposition.

Atomic concentrations of each element from the RECO system derived from XPS analysis, as presented in Table 2, allow for the determination of the stoichiometry of the analyzed samples. It is observed that the stoichiometry can be expressed as (Y_0.29_La_0.22_^Nd^_0.18_Sm_0.19_Gd_0.27_)CoO_3.43_, which is determined to exhibit a high degree of agreement with the theoretical formulation for a multicomponent RECO structure. This result provides strong evidence of the successful synthesis of the RECO material with the desired composition.

**Figure 3:**
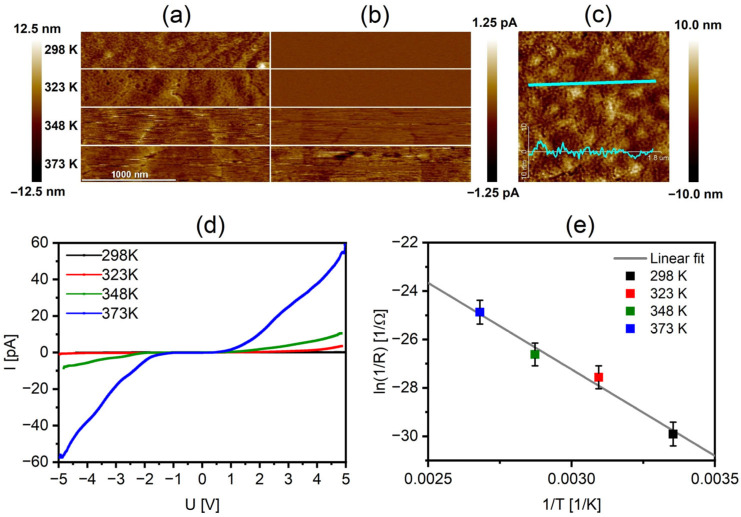
AFM images of the RECO on SiO_2_ thin film recorded at 298, 323, 348, and 373 K: (**a**) topography, (**b**) current distribution image, (**c**) cross-section profile (**d**) I−U plots in the range between–5 and 5 V, and (**e**) two-point probe method plots of ln(1/R) as a function of the reciprocal of the temperature (1/T) using the Arrhenius equation.

The conductivity of the RECO on a nanoscale amorphous quartz layer was investigated using atomic force microscopy operating in the PF-TUNA mode, allowing simultaneous gathering of the sample surface topography images along with its conductivity parameters, including current distribution image and current-voltage (I-U) plots. Conductivity measurements in the studied system are based on the electrical contact between the AFM probe and the conductive surface of the path [53,54]. It should be noted that the nature of the contact and the resulting conductivity parameters can be influenced by variations in surface roughness, resulting in different contact areas between the AFM tip and the sample surface. The AFM topography and current images were measured at room temperature (298 K) and the elevated temperatures of 323, 348, and 373 K. The graphical data in Figure 3a,b clearly demonstrate a significant increase in conductivity observed at the surface as the temperature applied to the thin film increases. The analysis of the cross-section profile presented in Figure 3c enabled the determination of the average surface roughness of the sample, yielding a value of 2.1 ± 0.4 nm, and it exhibited no significant changes with variations in temperature or electric current, indicating its thermal and electrical stability. Furthermore, the relatively small roughness of the thin film makes it non-susceptible to the common effect of measuring higher current at the contact areas of the measuring tip and its sides with the surface of the sample. This characteristic ensures that the mapped conductivity distribution remains relatively homogeneous and is unaffected by the surface non-uniformity effects, providing a balanced representation of the current distribution image. The corresponding I−U plots (Figure 3d) confirm the observed behavior, as the current values reach up to 1 nA for potentials above 4 V. The obtained current-voltage plots were used to calculate the activation energy (E_a_), which was found to be 1.23 ± 0.12 eV using the Arrhenius plot from Figure 3e.

**Figure 4:**
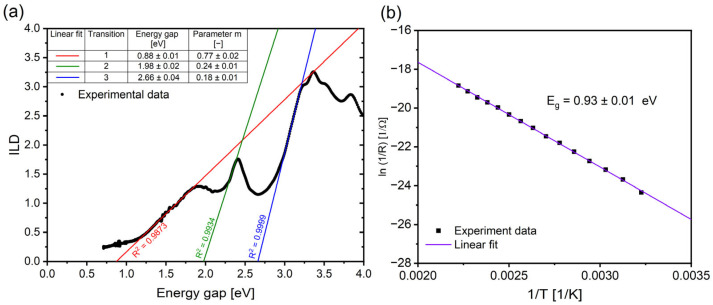
(**a**) the Inverse Logarithmic Derivative (ILD) of αhv as a function of photon energy hv with fitted straight lines for RECO/SiO_2_ and measured energy gap values and the transition parameter m. (**b**) calculated energy gap and plot of ln(1/R) as a function of the reciprocal of temperature (1/T) obtained using the Arrhenius equation derived from the four-point resistance method (FPRM).

The UV-VIS spectroscopic measurements were performed on the deposited RECO layer on the quartz substrate, and the measured absorbance of the thin layer was used to determine the energy gap of the semiconductor and the nature of its optical transitions. In this study, we implemented the Inverse Logarithmic Derivative (ILD) method to analyze the absorbance data for the RECO film deposited on the quartz substrate, according to the calculation method described in the reference [55]. The resulting graph (Figure 4a) exhibits three distinct regions, each corresponding to different optical transitions. The first is characterized by strong absorption, indicating the direct allowed transition of electrons from the valence band to the conduction band [56]. The energy gaps and the corresponding parameters m were then calculated from the graph, and the results are presented in Table 3. Analysis reveals that the RECO layer exhibits direct allowed transitions with a modified dispersion constant only for parameter m = 0.77 and the corresponding energy gap (E_a_) of 0.88 eV. Additionally, the presence of two other absorption peaks suggests the involvement of other types of transitions, including indirect allowed and direct/indirect forbidden transitions [57,58]. The observed phenomena could potentially arise from the nanometer thickness of the RECO semiconducting film and the refractive index of the quartz substrate and have direct implications for the design and optimization of the optoelectronic devices, both of which may potentially rely on the RECO thin film [59,60]. Further investigation of the underlying factors contributing to the modified dispersion constant or effective dimensionality could lead to a deeper understanding of the unique properties of the RECO thin film.

Figure 4b depicts a plot of the natural logarithm of the reciprocal of resistance (ln(1/R)) as a function of the reciprocal of temperature (1/T) derived from the four-point resistance method (FPRM). The energy gap was determined from the Arrhenius plot using the slope of the ln(R) versus (1/T) line, which was calculated using the Arrhenius equation [61]:

(3) $$E=\frac{k\;\ln(R_{0}/R_{1})}{\Delta (1/T)}$$

where E represents the energy gap, k is the Boltzmann constant, R_0_ and R_1_ are the resistances at the initial and final temperatures, and Δ(1/T) is the change in the reciprocal of temperature. The energy gap values obtained for the RECO thin film system exhibit relatively consistent results across different measurement methods, differing by no more than 30%. In particular, the energy gaps obtained from the AFM U−I, FPRM, and UV/VIS methods were found to be 1.23, 0.93, and 0.88 eV, respectively. These findings indicate that RECO thin film may be a promising candidate for optoelectronic and electrocatalyst devices requiring narrow energy gap values. Examples of such applications include oxygen evolution catalysis [62], low-energy light-emitting diodes [63,64], or near-infrared photodetectors [65]. The combination of a narrow energy gap with relatively good visible light adsorption may allow the RECO thin films to become a promising component to consider for solar cell application [66]. Furthermore, the observed increase in conductivity with rising temperatures indicates that the energy carriers play an increasing role in the optical transition of the RECO thin film [67]. These observations highlight the importance of understanding the modified dispersion constant or effective dimension of the material in affecting both the electrical and optical properties of potential RECO-based electronic devices. Further investigation of these aspects is essential to gain deeper insights and facilitate the development of the potential optimized RECO-based electronic devices.

## 4. Conclusions

In this study, the RECO thin film was successfully deposited on the amorphous SiO_2_ substrate. Structural and chemical analysis revealed that the thin film with an orthorhombically distorted (*Pnma*) crystal lattice is consistent with the bulk material. The film was found to be composed of Y, La, Nd, Sm, Gd, Co, and O elements with stoichiometry consistent with the theoretical composition. Additionally, the presence of a Co element in both Co^2+^ and Co^3+^ oxidation states has been confirmed, while the rare-earth elements were found to be in their trivalent states. Electronic and optical measurements revealed activation energy values of 1.23 eV (AFM U-I), 0.93 eV (FPRM), and 0.88 (UV/VIS), respectively. The measured values categorize the RECO thin film as a narrow gap semiconductor material and indicate that it exhibits distinct electronic and optical properties compared to its bulk form, thus enabling the RECO material to be susceptible to tuning the energy gap value by nanostructuring or introducing local strain in thin film fabrication. Furthermore, the notable narrow energy gap of RECO, coupled with its relatively good absorption in the visible spectrum, makes it a compelling candidate for further investigation for applications in low-energy infrared optical devices, electrocatalysis of the oxygen evolution reaction, and efficient light energy storage in solar cells. Further studies are needed to explore the potential applications of the RECO thin film and to optimize the deposition process to improve and tune its structural and optoelectronic properties. Nevertheless, the successful deposition and characterization of the RECO thin film provide a basis for future research on the properties and potential applications of this material.

## Figures and Tables

**Table 1:** XRR results calculated for RECO thin film on a quartz substrate.

Layer	Density [g cm^−1^]	Thickness [nm]	Roughness RMS [nm]
quartz substrate	2.6 ± 0.2	-	1.6 ± 0.2
RECO thin film	8.0 ± 0.9	59.0 ± 0.5	2.4 ± 0.4

**Table 2:** Surface composition (at-%) determined by XPS after pre-sputtering the surface with a 2 kV Ar+ ion beam.

Element	O	Co	Y	La	Nd	Sm	Gd
	at-%						
Surface	61.1 ± 6.4	17.8 ± 2.1	5.3 ± 0.2	4.0 ± 0.3	3.3 ± 0.3	3.4 ± 0.2	4.8 ± 0.3
Theoretical	60.0	20.0	4.0	4.0	4.0	4.0	4.0

**Table 3:** Energy gap from different methods.

Method	Energy Gap [eV]	R^2^ [−]
AFM U-I	1.23 ± 0.12	0.9810
FPRM	0.93 ± 0.01	0.9995
UV/VIS	0.88 ± 0.02	0.9873
	1.98 ± 0.04	0.9934
	2.66 ± 0.07	0.9899

## Data Availability

The data that support the findings of this study are available from the corresponding authors upon reasonable request.
